# Editorial: Systems Biology of Hosts, Parasites and Vectors

**DOI:** 10.3389/fcimb.2021.796475

**Published:** 2021-11-04

**Authors:** Luiz Gustavo Gardinassi, Sandra Regina Maruyama, Cinzia Cantacessi

**Affiliations:** ^1^Institute of Tropical Pathology and Public Health, Federal University of Goiás, Goiânia, Brazil; ^2^Department of Genetics and Evolution, Center for Biological Sciences and Health, Federal University of São Carlos, São Carlos, Brazil; ^3^Department of Veterinary Medicine, University of Cambridge, Cambridge, United Kingdom

**Keywords:** genome, transcriptome, proteome, microbiome, vector interaction, control, drug resistance, evolution

Parasites cause significant morbidity and mortality in humans, livestock, and companion animals, imposing major challenges for public and veterinary health, and the food industry, worldwide. Parasitic diseases are influenced by a wide range of factors that add complexity to the understanding of the host-parasite-vector triad. A thorough knowledge of parasite biology, and of the interactions between these pathogens and their vertebrate and/or invertebrate hosts is key to discover and develop novel and sustainable strategies to reduce infection burden and assist elimination.

Systems Biology has emerged to provide an integrated overview of biological systems on the “post-genomic era” and predict how they operate to produce a determined phenotype (Ideker et al., 2001; Chuang et al., 2010). Omics technologies coupled to computational biology have been applied to study different aspects of parasites (Bourgard et al., 2018; Maruyama et al., 2019b; Garcia et al., 2020), host responses (Saric et al., 2010; Gardinassi et al., 2016; Gardinassi et al., 2018; Hargrave et al., 2019; Maruyama et al., 2019a), interaction with vectors (Srivastava et al., 2016; Inbar et al., 2017) and the host microbiome (Jenkins et al., 2018; Cortés et al., 2020; Jenkins et al., 2021). Considering these concepts and philosophy, this Research Topic was formulated to communicate high-quality work in this area of investigation and associated disciplines, which gathered original research, review, perspective and hypothesis and theory articles.

The first published article of this Research Topic tackled a technical issue related to the zoonotic protozoan parasite, Giardia *lamblia*. Heller et al. applied shotgun mass spectrometry to uncover that transfection of the reference strain WBC6 with glucuronidase A (GusA) from *Escherichia coli* affects the cellular proteomic profile and induces antigenic variation. Results from this study have strong implications for the interpretation of experiments involving transfected *Giardia* and related controls, because comparisons with un-transfected trophozoites may lead to spurious results.

Other protozoan parasites including *Leishmania* and Plasmodium were also addressed. Horácio et al. provided a perspective of how systems biology approaches have been used and can contribute to future discoveries in drug resistance of *Leishmania* parasites. Current therapeutics and how networks of biochemical pathways can be involved in drug resistance were explored, with the authors indicating antioxidant defense enzymes as biomarkers of resistance and drug targets.

The development of new therapeutic venues or vaccines to prevent disease can be explored with systems biological approaches, including immunopeptidomics. In this Research Topic, Juanes-Velasco et al. reviewed advances and strategies used to identify immunogenic peptides presented on human leukocyte antigen (HLA) molecules and recognized by T cells. The review is broad and provides an informative overview of immunopeptidomics in infectious diseases and cancer. We highlight the discussion of the potential application of this technology to identify *Plasmodium falciparum* antigens to compose improved vaccines against malaria.

Other strategies have focused on the identification of targets blocking transmission of *Plasmodium* to their vectors (Gonçalves and Hunziker, 2016). In This Research Topic, Niu et al. have analyzed transcriptomics data of *P. falciparum* to study genes coding for proteins that interact with the vector midgut. The authors identified 6 proteins interacting with vector midgut lysate, with further experiments suggesting that Pfs16 might interact with the vector midgut, and this might represent a target to block transmission.

Addressing aspects of helminth parasites, Fontenla et al. used bioinformatics approaches to analyze and compare small non-coding RNA pathways in Platyhelminthes. The authors observed that Piwi proteins, which interact with and regulate piRNA, are conserved in free-living but absent in parasitic flatworms, which could potentially influence the divergence to parasitism in flatworms.

Genomic diversification and evolution of helminths have led to the emergence of defense strategies, some of which favor parasitism, such as enzymatic antioxidant systems (Chiumiento and Bruschi, 2009). In this Research Topic, Dorey et al. discussed genomic, transcriptomic, and proteomic analyses contributing to the emerging concept of non-antioxidant roles of thioredoxin-1 and peroxiredoxin-1 of the fluke *Fasciola hepatica*. The authors propose these proteins operate mostly *via* immunomodulatory mechanisms rather than the stress-inducible thiol-dependent cascade.

A Hypothesis and Theory article by da Costa et al. discusses carcinogenic- helminthiasis, chemical carcinogenesis, and therapeutic strategies to control helminths and associated pathology. The authors focused on carcinogenic potential of urogenital schistosomiasis and suggested that selected parasite metabolites could interact with host DNA and inhibit repair. Many other factors can be implicated in this process, that systems biology tools might contribute to discover.

Animal models of helminthic diseases are critical to understand both parasite development in hosts and evasion mechanisms, as well as the dynamics of the mammalian immune response to these parasites. In this Research Topic, Montaño et al. compared and discussed the proteomes of *Nippostrongylus brasiliensis*, *Heligmosomoides polygyrus bakeri* and *Trichuris muris*, all important models of human helminthiases, and argue that these parasites represent key sources of potential vaccine candidates and novel immunomodulatory molecules.

In this Research Topic, Rosa et al. analyzed the gut microbiome of humans infected with *Trichuris trichiura* and compared it with that of mice infected with *T. muris*. Besides demonstrating the applicability of this mouse model to study whipworm infection, the authors identified bacterial taxa whose dynamics were associated with infection in mice and humans, as well as with anthelmintic treatment, and suggested that the genera *Escherichia* and *Blautia* might represent promising targets for future mechanistic studies of host-parasite-microbiome interactions.

The impact of *Calicophoron daubneyi*, a parasite of ruminant livestock over the rumen microbiome was investigated by Allen et al.; the authors described 378 proteins in extracellular vesicles (EVs) released by *C. daubneyi via* proteomic analyses. Furthermore, parasite EVs modulated total bacteria concentrations in rumen, suggesting a role for parasite EVs in worm-microbiome interactions and a possible role of these vesicles in the establishment and maintenance of fluke infections.

Overall, the articles included in this Research Topic covered intriguing questions related to protozoans and helminths, and their interaction with vectors, hosts and associated microbiome, control strategies, drug resistance, as well as diversification and evolution. These studies contribute to a better understanding of parasites and the pathological processes they cause, while stimulating further research in the field.

## Author Contributions

LG wrote the editorial draft. SM and CC revised the editorial. All authors approved the final version of the manuscript.

## Funding

This work was supported by the Serrapilheira Institute (grant number Serra – R-2011-37433) to LG. SRM is a Young Investigator awardee and received a fellowship from São Paulo Research Foundation (FAPESP 2017/16328-6).

## Conflict of Interest

The authors declare that the research was conducted in the absence of any commercial or financial relationships that could be construed as a potential conflict of interest.

## Publisher’s Note

All claims expressed in this article are solely those of the authors and do not necessarily represent those of their affiliated organizations, or those of the publisher, the editors and the reviewers. Any product that may be evaluated in this article, or claim that may be made by its manufacturer, is not guaranteed or endorsed by the publisher.
